# CNN-based framework using spatial dropping for enhanced interpretation of neural activity in motor imagery classification

**DOI:** 10.1186/s40708-020-00110-4

**Published:** 2020-09-03

**Authors:** D. F. Collazos-Huertas, A. M. Álvarez-Meza, C. D. Acosta-Medina, G. A. Castaño-Duque, G. Castellanos-Dominguez

**Affiliations:** 1 Signal Processing and Recognition Group, Universidad Nacional de Colombia, Manizales, Colombia; 2 Cultura de la Calidad en la Educación Research Group, Universidad Nacional de Colombia, Manizales, Colombia

**Keywords:** Motor imagery, Convolutional Neural Networks, Spatial dropping

## Abstract

Interpretation of brain activity responses using motor imagery (MI) paradigms is vital for medical diagnosis and monitoring. Assessed by machine learning techniques, identification of imagined actions is hindered by substantial intra- and inter-subject variability. Here, we develop an architecture of Convolutional Neural Networks (CNN) with an enhanced interpretation of the spatial brain neural patterns that mainly contribute to the classification of MI tasks. Two methods of 2D-feature extraction from EEG data are contrasted: Power Spectral Density and Continuous Wavelet Transform. For preserving the spatial interpretation of extracting EEG patterns, we project the multi-channel data using a topographic interpolation. Besides, we include a spatial dropping algorithm to remove the learned weights that reflect the localities not engaged with the elicited brain response. We evaluate two labeled scenarios of MI tasks: bi-class and three-class. Obtained results in an MI database show that the thresholding strategy combined with Continuous Wavelet Transform improves the accuracy and enhances the interpretability of CNN architecture, showing that the highest contribution clusters over the sensorimotor cortex with a differentiated behavior of rhythms $\mu$ and $\beta$.

## Introduction

The motor imagery (MI) paradigm is a form of brain–computer interface (BCI) that performs the imagination of a motor action without real execution, relying on the similarities between imagined and executed actions at the neural level. MI is usually measured with electroencephalography (EEG) to register brain activity on the scalp surface. Thus, assessment and interpretation of MI brain dynamics in the sensorimotor cortex may contribute to applications ranging from evaluation of pathological conditions and rehabilitation of motor functions [1, 2], motor learning and performance [3], improving the learning of different abilities [4], among others. In education scenarios, the Media and Information Literacy methodology proposed by the UNESCO covers several competencies that are vital for people to be effectively engaged in all aspects of human development [5]. Nevertheless, one of the main challenges in implementing MI practice is recognizing and identifying the imagined actions since EEG signals have substantial intra- and inter-subject variability [6].

Currently, there is an increasing interest in deep learning models that are composed of multiple processing layers of inference using data representations with multiple levels of abstraction. In discriminating physiological signals, Convolutional Neural Networks (CNN) become the leading deep learning architectures due to their regularization structure and degree of translation invariance [7], yielding an outstanding ability in transferring knowledge between apparently different tasks of classification [8, 9]. Thus, CNN models are useful in learning features related to brain imaging and neuroscience discovery [10]. Nevertheless, for applications in MI tasks, designing an available end-to-end CNN architecture remains a challenge due to several restrictions: their large number of hyperparameters to be learned increase the computational burden (being unsuitable for online processing [11]), and complicated multilayer integration to encode relevant features at every abstraction level of the input EEG data [12].

Another unresolved issue is the interpretability of results provided by CNN models [13]. That is, along with the improved accuracy, the learned features can be hard to understand within the context of the original MI paradigm. The value of neural activity interpretation becomes evident in purposes like a medical diagnosis, monitoring, and computer-aided learning [14]. As a tool in image processing, CNN architecture has been discussed for enhancing the physiological explanation of MI paradigms represented by multiple time-series (time dimension), which reflect the brain responses across the sensorimotor cortex (spatial dimension), and commonly related to $\mu$ and $\beta$ rhythms (spectral dimension). For representing local and global structures in CNN models, therefore, the extraction of time-series features is increasingly realized as a multi-dimensional tensor that retains the EEG data structure throughout the learning process, by adequately encoding the spatio/spectro-temporal relationships of the measured MI responses [15]. Nevertheless, CNN models should extract the structure of multi-dimensional images properly to preserve the domain information of interest. Intending to make the learned features more interpretable in MI tasks, two main aspects are to be considered to retain the spatial locality of CNN models: (i) improving the 2D-feature extraction from EEG data for feeding the CNN models, and (ii) enhancing the image-based EEG representation to integrate spatial domain knowledge with the extracted 2D spectro-temporal features.

For building 2D-maps in discrimination of MI tasks, several algorithms of feature extraction are employed in CNN models, including the following: common spatial patterns due to the high recognition rate and computational simplicity [16]; event-related synchronization to capture the channel-wise temporal dynamics of the power signal [17]; empirical mode decomposition to deal with EEG nonstationarity [18, 19]; and time–frequency planes using the Fourier and wavelet transforms are frequently extracted because they allow a more straightforward interpretation [20–23], the latter decomposition being better suited to deal with sudden changes in EEG signals. Nonetheless, the extracted 2D images tend to have substantial variability in patterns across trials due to inherent nonstationarity, artifacts, a poor signal-to-noise ratio of EEG signals, individual differences in cortical functioning (like subjects exhibiting activity in different frequency bands).

Concerning the integration of electrode montages with the extracted 2D features, topographical representations are applied, involving either local or global spline techniques to interpolate the spatial distribution of the potential field on the scalp from distributed electrode arrays. For low electrodes distributions, adequate mapping is the spherical spline interpolation [24]. One strategy of integration is to incorporate prior knowledge to optimize the neural network structure for handling the lack of significant samples in smaller datasets. For instance, pre-trained networks are used, but assuming a substantial similarity between pre-training and target sets [25–27]. Otherwise, some ambiguity may remain in the foolproof nature of the pre-trained network methodology [28]. In the case of MI tasks, there are very few accessible datasets having some differences in implementing the paradigm. Another integration approach is to have some form of spatial dropping algorithm to remove candidate localities known to be not engaged with the elicited brain response. Relying on the fact that motor imagery responses are directly related to electrocortical activity over the sensorimotor area, the spatial dropping can be performed either subject-independent by excluding all electrodes out of the motor cortex before training and validation [29–31], or by thresholding the electrode contribution after training and validation for each subject.

Here, we develop a CNN architecture with an enhanced interpretation of the spatial activity of brain neural patterns that mainly contribute to the classification of MI tasks (left, right hand, and foot). Following the approach developed by [32], the CNN framework is designed, for which we validate two commonly used techniques of feature extraction from EEG data: power spectral density and continuous wavelet transform. For preserving the spatial interpretation of extracting EEG patterns, we project the multi-channel data using a topographic interpolation. Besides, we include a spatial dropping algorithm to remove the learned weights that reflect the localities not engaged with the elicited brain response. Obtained results in a MI database show that the thresholding strategy is desirable since the highest contribution clusters over the sensorimotor area with differentiated behavior between $\mu$ and $\beta$ bands. The present paper’s agenda is as follows: Section 2 describes the collection of MI data used for validation. Besides, it presents the fundamentals of feature extraction of time–frequency (*t-f*) EEG patterns and describes the design of Convolutional Neural Networks, including the spatial dropping strategies for motor imagery classification. Further, Section 3 provides a summary of the classifier accuracy performed by the extracted *t-f* vectors and evaluates the interpretability of learning weights for distinguishing between MI tasks. Lastly, Section 5 gives critical insights into the performed interpretation and accuracy, and address some limitations and possibilities of the presented CNN-based framework.

## Materials and methods

Description of MI database and preprocessing. We perform experimental validation with nine subjects ($$N_S = 9$$) of Dataset 2a^1^, holding EEG signals acquired from the scalp by a *C*-channels montage ($$C = 22$$). Each raw EEG channel $${\varvec{x}}^c{\in }\mathbb {R}^{T}$$ was sampled at 250 Hz (i.e., at sample rate $$\varDelta t{=}0.004$$ s) and passed through a five-order bandpass Butterworth filter within $$\varOmega = [8,30]$$ Hz. Since earlier works have shown that electrical brain activities prompted by motor tasks are frequently related to $$\mu $$ and $$\beta $$ rhythms [33], the spectral range is split into the following bandwidths of interest: $$\varDelta f{\in }\,$$$$ \{ \mu {\in }\, [8{-}12], $$$$\beta _{{\text {low}}}{\in }\,[16{-}20], $$$$\beta _{{\text {med}}}{\in }\,[20{-}24],$$$$ \beta _{{\text {high}}}{\in }\,[24{-}28] \}$$ Hz.

For performing an MI task, each trial began with an acoustic cue “beep” (at 0 s), and along with a fixation cross appeared on the black screen. After 2 s (at 2 s), an arrow cue appeared for 1.25 s on the screen, pointing in one direction according to the evaluated MI task: the left (left hand), right (right hand), or down (foot). The subjects were then instructed to image the corresponding imaginary movement between 3 s and 6 s. At 6 s, the screen was black again, allowing the subjects to relax. Then, each subject performed a run of each MI task while the cross re-appeared within the time interval, starting from 3.25 to the recording end, *T* s. The recordings were collected in six runs separated by short breaks, performing $N_\lambda = 72$ trials per class and each one lasting $T=7$ s. We validated two labeled scenarios: bi-class (left hand and right hand), and three-class (left hand, right hand, and foot). Testing is carried out using only the labeled trials with the removed artifacts.

Feature extraction of t-f EEG patterns. In the first case, the feature set is extracted from the Fourier decomposition method. So, provided the EEG sample frequency $F_s{\in }\mathbb {R}^{+}$, the power spectral density (PSD) vector ${\varvec{s}} = \{s_f{\in }\mathbb {R}^{+}:f{\in }N_B\}$, with $N_B = l\lfloor F_s/2\rfloor$, is estimated through the nonparametric Welch’s method that calculates the fast Fourier transform (FFT) algorithm on a set of $M{\in }\mathbb {N}$ overlapping segments, which are split from the preprocessed EEG data vector ${{\varvec{x}}^c}$. Due to the non-stationary nature of EEG data, the piecewise stationary analysis is carried out over the set of the extracted overlapping segments that are windowed by a smooth-time weighting window ${\varvec{\alpha }}{\in }\mathbb {R}^\tau$ that lasts $\tau {\in }\mathbb {N}$ ($\tau <T$), yielding a set of the time segments $\{{{\varvec{v}}}^{m}{\in }\mathbb {R}^\tau : m{\in }M\},$ where $v^m_t{\in }\mathbb {R}$ ($t{\in }\tau$) is *t*th element of ${\varvec{v}}^{m}$. So, the *t-f* patterns are extracted from EEG signals through the modified periodogram vector, ${\varvec{u}} = \{u_f{\in }\mathbb {R}^+\}$, ${\varvec{u}}{\in }\mathbb {R}^{N_B}$, computed as follows:

1 $$\begin{aligned} u_f = \mathbb {E}\left\{ \big |\sum \limits _{t\in \tau }{{{v}^{m}_t} \exp {( {-j{2\pi t f }} )}}\big |^2:\forall m\in M\right\} . \end{aligned}$$

Thus, the resulting PSD vector is computed with spectral components defined as ${s }_f = {u_f}/({M\nu }),$ being $\nu = \mathbb {E}\left\{ |\alpha _t|^2:\forall t{\in }\tau \right\} ,$ and $\mathbb {E}\left\{ \cdot \right\}$—the expectation operator.

In the second case, the feature set is extracted from Continuous Wavelet Transform (CWT) that quantifies similarity between a given equally sampled time-series at time spacing $\delta _{t}{\in }\mathbb {R}$ and a previously fixed base function $\psi \left( \eta \right)$, termed *mother wavelet* ruled by a dimensionless parameter vector $\eta {\in }\mathbb {R}$. Namely, each time element of the CWT vector ${\varvec{{\varsigma }}}^g{\in }\mathbb {C}^T$ is extracted from the preprocessed EEG time-series ${\varvec{z}}{\in }\mathbb {R}^{c}$ at scale $g{\in }\mathbb {R}$ by accomplishing their convolution with the scaled and shifted mother wavelet in the form:

2 $$\begin{aligned} \varsigma _t^f= \sum \limits _{\tau \in T}{z_{\tau } \psi ^*\left( {(\tau -t)}\delta _t,{f}\right) }, \end{aligned}$$

where notation $(^{*})$ stands for the complex conjugate.

To build a picture showing amplitude variations through time in Eq. (2), both procedures of wavelet scaling *g* and translating through the localized time index $t{\in }T$ are used. As a result, the extracted wavelet coefficients provide a compact representation pinpointing EEG data’s energy distribution in time and frequency domains. Therefore, the resulting CWT vector is computed with spectral components defined as ${s}_f = \mathbb {E}\left\{ \varsigma _t^f:\forall t{\in }\tau \right\}$.

Having extracted the feature set, we further compute a real-valued representative vector, ${\varvec{\rho }}^{r,\varDelta f} {\in }\mathbb {R}^{C}$ for each trial $r{\in }R$, with electrode elements that accumulate the spectral contribution as follows:

3 $$\begin{aligned} {\rho }^{r,c} = {\sum \limits _{\eta _{\min } {\le } f {\le }\eta _{\max }}{ {s}^{r,c}_{f} } } ,\, f\in \varDelta f, \end{aligned}$$

where the frequencies $\eta _{\min }$ and $\eta _{\max }$ determine each one of the bandwidths of interest $f{\in }\varDelta f$, respectively, within the most discriminating MI information is assumed to be concentrated.

Then, we map the multi-channel data per patient on a 2D surface, aiming to preserve the spatial interpretation of the extracted *t-f* patterns. In order to preserve the distance between electrodes in the 3D plane, we compute the topographic interpolation matrix across all trials, $\{ {\varvec{S}} ({\varvec{\rho }}^{r,\varDelta f}){\in }\mathbb {R}^{S\,{\times }\, S'}:\forall r{\in }R\}$, through the projecting matrix that maps each EEG trial field, ${\varvec{\rho }}^{r,\varDelta f}$, as a 2-D circular view (looking down at the head top) using spherical splines that sizes $(S\,{\times }\, S')$^2^, as detailed in [34].

Motor imagery classification using Convolutional Neural Networks. The proposed CNN architecture contains three learning stages: (i) convolutional layer that holds a set of kernel filters, $\{{\varvec{K}}_i{\in }\mathbb {R}^{K\,{\times }\, K}:i{\in }I\}$ (*I* is the number of used kernel filters), together with the corresponding bias vectors $\{{\varvec{b}}_i{\in }\mathbb {R}^{SS'}\}$, which are applied by a sliding window across each topographic map ${\varvec{S}}({\varvec{\rho }}^{r,\varDelta f})$, yielding the convolution feature map as below:

4 $$\begin{aligned} {\varvec{\varXi }}^{r,i,\varDelta f}=\gamma _1\left( {\varvec{K}}_i\otimes {\varvec{S}}({\varvec{\rho }}^{r,\varDelta f}) +{\varvec{b}}_i\right) ,\, {\varvec{\varXi }}^{r,i,\varDelta f}{\in }\mathbb {R}^{S\,{\times }\, S'}, \end{aligned}$$

where $\gamma _1(\cdot )$ is a non-linear activation function, and $\otimes$ denotes the convolution operator. Of note, a zero-padding method is adopted to prevent losing the feature dimension, so that the output and input sizes of convolution mapping can be the same after the zero-padding procedure.

(ii) Pooling layer that is a down-sampling stage to reduce the dimension of output neurons in ${\varvec{\varXi }}^{r,i,\varDelta f}$ through a pool operator matrix ${\varvec{\bar{K} }}{\in }\mathbb {R}^{K'{\times }K'}$, with $K'\le K,$ aiming at decreasing the computational burden and the over-fitting issue. Then, each down-sampled map ${\varvec{\bar{\varXi }}}^{r,i,\varDelta f}$ is rearranged into a vector form ${\varvec{\bar{\xi }}}^{r }{\in }\mathbb {R}^{G G'IN_f}$ (with $G\le S,G'\le S'$) by concatenating all matrix rows across $\varDelta f$ and *i* domains.

(iii) A fully connected stage that includes a neural network with all neurons ${\varvec{h}}^r(q){\in }\mathbb {R}^{N_h(q)}$ connected directly to the outputs of preceding layer $q{-}1$ as follows:

5 $$\begin{aligned} {\varvec{h}}^{r}(q)=\gamma _2\left( {\varvec{W}}(q){\varvec{h}}^{r}(q-1)+{\varvec{\beta }}(l)\right) ,\, q=\overline{2,Q}, \end{aligned}$$

where ${\varvec{h}}^{r}(1)\, =\, {\varvec{\bar{\xi }}}^{r}$, ${\varvec{W}}$, sizing ${G G'IN_f{\times }N_h(q)}$, is the weighting matrix that contains the connection weights between the preceding neurons and the hidden units $N_h$ of layer *q*, ${\varvec{\beta }} (q){\in }\mathbb {R}^{N_h(q)}$ is the bias neuron, and $\gamma _2(\cdot )$ is an activation function.

As a result, we obtain the output vector set $\{{\varvec{y}}^{r} = {\varvec{h}}^{r}(Q)\},$ with ${\varvec{y}}^{r}{\in }[0, 1]^{N_\lambda }$, representing $N_\lambda$ mutually exclusive classes, so that the last layer is tied to the output dimension ($N_h = N_\lambda$).

Due to the CNN-model training back-propagates the discriminating information, through the tied weights, from the hidden spaces in the input data, we propose to assess the relevance of input feature mappings, employing the matrix ${\varvec{W}}(q){\in }\mathbb {R}^{D{\times }N_h}$ that holds the row vectors ${\varvec{w}}_d^{q}{\in }\mathbb {R}^{N_h}$ with $D{=}G G'IN_f$. Based on the fact that each ${\varvec{w}}_d^{q}$ measures the contribution of input features to build the hidden space ${\varvec{h}}^{r}(q)$, the relevance of *d*-th feature is assessed as the generalized mean of its corresponding reverse projection vector, that is, $\varrho _d^{q} = \Vert {\varvec{w}}_d^{q}\Vert _{p}$, yielding the vector ${\varvec{\varrho }}^{q} = \{\varrho _{d}^{q}{\in }\mathbb {R}^{+}; \forall d{\in }D\}$, where notation $\Vert \cdot \Vert _p$ stands for ${l}_p$-norm. The obtained relevance vector ${\varvec{\varrho }}^{q}$ is reshaped into an estimated feature mapping matrix $\widetilde{{{{\varvec{\varTheta }}}}} {\in }\mathbb {R}^{S{\times }S'}$ that is computed for each $\varDelta f$ as follows:

6 $$\begin{aligned} \widetilde{{{{\varvec{\varTheta }}}}} = \phi \left( \mathbb {E}\left\{ \widetilde{{\bar{{\varvec{\varXi }}}}}_i:\forall i{\in }I\right\} \right) , \end{aligned}$$

where $\widetilde{{\bar{{\varvec{\varXi }}}}}_i{\in }\mathbb {R}^{G{\times }G'}$ is the reconstructed feature mapping for *i*-th kernel filter, and $\phi (\cdot )$ is an extrapolation operator that maps from $G{\times }G'\rightarrow S{\times }S'$. In this way, the obtained $\widetilde{{\varvec{\varTheta }}}$ highlights the spatial discriminative information projected from topographic maps.

## Experiments

We validate the proposed CNN-based MI classification framework by appraising the following procedures: (i) preprocessing and extraction of *t-f* planes, evaluating the extraction methods of power spectral density and continuous wavelet transform, for which the corresponding parameter tuning is carried out; (ii) tuning of CNN architecture for MI discrimination, evaluating the spatial dropping algorithm proposed for preserving the interpretation of the extracted 2-D features. Two approaches for dropping are appraised: removing all electrodes out of the sensorimotor area before training and validation, and thresholding the electrode contribution after training and validation.

Extraction of t-f feature patterns. Each channel recording, ${\varvec{x}}^{c}{\in }\mathbb {R}^{T},$ is split into $N_\tau = 5$ segments, $\{{\varvec{x}}^{c}{\in }\mathbb {R}^{\tau },\, \tau <T\}$, using a sliding window approach with a segment length $\tau = 2$ s with overlap $\delta \tau = 1$ s. Within each segment ${\varvec{x}}^{c}$, PSD estimates are computed, fixing the following parameters: $\tau = 256, {\delta _\tau = 0.9\tau }$. Likewise, we compute the CWT vector ${\varvec{{\varsigma }}}^g$, selecting the Morlet wavelet as $\psi$ that is frequently used in spectral analysis of EEG signals [35]. So, we extract the continuous wavelet coefficients within each time segment using a complex Morlet wavelet, adjusting the scaling value to $g = 16$ and the sampling period to $1/\varDelta t$.

For either method of feature extraction, we perform validation in four different scenarios for spectral bandwidths of interest $f{\in }\varDelta _f$: *A*) $\mu$, *B*) $\beta$, *C*) $\mu \cup \beta$, and *D*) $\mu \cup \beta _{{\text {low}}}\cup \beta _{{\text {med}}}\cup \beta _{{\text {high}}}$.

**Fig. 1:**
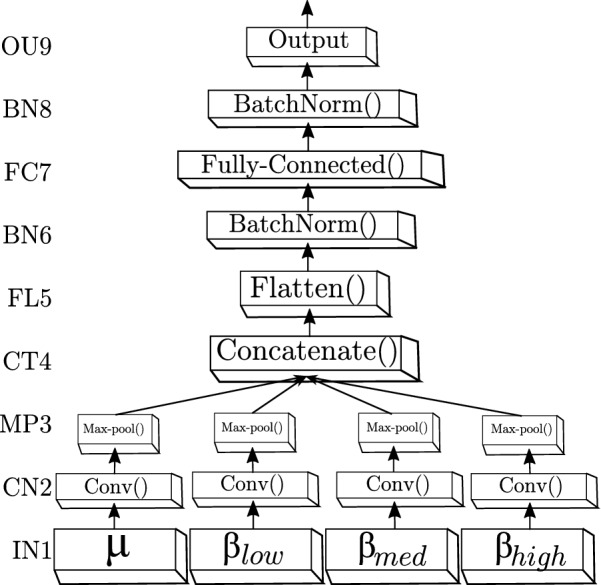
The proposed CNN structure that is based on *Wide&Deep* neural network handling multiple inputs. The first layer (IN1) is the input, the second (CN2) and third layers (MP3) are hidden and accomplish the feature mapping generation, while the next block (ranging from the output of layer CT4 to the OU9 layer) comprises the classification stage

Proposed CNN architecture for MI discrimination. The adopted multiple input CNN model is based on the non-sequential *Wide&Deep* neural network [36] that performs learning of deep patterns (using the deep path) under simple rules (through the short path), having the following units (Fig. 1):

- **IN1**: Input layer that holds an image set sizing $42\, {\times }\, 56$.
- **CN2**: Convolutional layer (first hidden layer). We use two spatial filters that perform two resulting feature maps, sizing $42 \,{\times }\, 56$. Each convolution kernel has a size of $3 \,{\times }\, 3$, using a stride of one sample. In addition, this layer incorporates a rectified linear unit *ReLU* through the activation function $\gamma _1(\cdot )$ [37].
- **MP3**: Max-pooling layer (second hidden layer). This layer sub-samples the resulting mapping that picks up the maximum value of each feature map to reduce the number of output neurons, also using a stride of one sample. Thus, each feature mapping in **CN2** is down-sampled using a pool size of $2 \,{\times }\, 2$, resulting in a matrix of size $21 \,{\times }\, 28$.
- **CT4**: Concatenate layer, linking together of all resulting MP3 feature maps into a single block.
- **Fl5**: Flatten layer that arranges the set of concatenated feature maps from CT4 into a single 1D array. So, the map is vectorized into a one-dimensional array of size $(21)(28)(2)(4) = 1176$ points, resulting from 2 spatial filters, and 4 bandwidths of interest.
- Batch normalization (BN) layers (**BN6** and **BN8**) that address the vanishing and exploding gradient problems presented in fully connected networks. To cope with this issue, all inputs of the previous layer at each batch are zero-scored, holding the mean activation close to 0 and the activation standard deviation close to 1.
- **FC7**: Fully connected layer (third hidden layer) that is linked to each neuron of **OU9**, holding $h_u$ neurons for which the weight values are regularized through the parameters ($l_1$, $l_2$) using the Elastic Net regularization. According to [38], Elastic Net is used for preventing over-fitting by penalizing a model having large weights, and can be used more naively, e.g., when little prior knowledge is available about the dataset. This layer uses a rectified linear unit *ReLU* as the activation function $\gamma _2(\cdot )$. The following parameter setting of **FC7** is fixed:

- Number of neurons are fixed through an exhaustive grid search within $h_u = [50,100,\ldots ,550]$.
- The learning rate is fixed at $lr = \exp ({-3})$.
- The optimizer used is the *Adam algorithm* and the *loss function* used is the mean squared error (MSE).
- The regularization parameters $l_1$ and $l_2$ are tuned by a grid search around [0.001, 0.01, 0.1].

- **OU9**: Output layer having two or three neurons, each one representing either task label (left hand, right hand or foot). This layer is fully connected to **FC7** and uses the *softmax* procedure as the activation function $\gamma _2(\cdot )$.

Evaluating metrics of classifier performance. As a measure of performance, the classifier accuracy $a_c{\in }\mathbb {R}[0,1]$ is computed as follows:

7 $$\begin{aligned} a_c =\frac{T_P + T_N}{T_P + T_N + F_P + F_N}, \end{aligned}$$

where $T_P$, $T_N$, $F_P$ and $F_N$ are true-positives, true-negatives, false-positives, and false-negatives, respectively.

Besides, the kappa value, $\kappa {\in }\mathbb {R}[0,1]$, is computed to evaluate the accuracy performance when removing the impact of random classification as follows [39]:

8 $$\begin{aligned} \kappa = ({a_c-p_e})/({1-p_e}),\, \end{aligned}$$

where $p_e = 0.5$ for bi-label problems.

A cross-validation scheme is performed to evaluate CNN-based classifier performance. Thus, the set of training trials per subject is randomly partitioned using a stratified tenfold cross-validation to generate the set of validation trials. This procedure is repeated ten times by shifting the test and training dataset.

## Results

Performed bi-class accuracy of extracted t-f planes. Initially, we discuss the classifier performance of the computed PSD vectors of contribution, ${\varvec{\rho }}^{r,\varDelta _f}$. In each one of the tested scenarios for spectral bandwidths of interest, parameter tuning is carried out to reach the maximum accuracy within the MI interval $[3{-}5]$ *s*. As seen in Table 1, the use of only one rhythm ($\mu$ or $\beta$) is not sufficient to reach the best values of accuracy. Moreover, the $\beta$ waveform drops to $80\%$. Their combination $\mu \cup \beta$ barely helps the classifier rule. Thus, the last validating scenario (i.e., *D*) reaches the best performance on average across all subjects, meaning that the inclusion of more detailed information of $\beta$ sub-bands allows improving the accuracy of PSD vectors. Concerning the individual performance, the subjects A02T, A01T, A04T, and A05T achieve the lowest values, while A08T, A09T, and A03T accomplish the best results. Regarding the CWT-based contribution vectors, the bottom part of Table 1 shows that the use of every spectral bandwidth scenario allows enhancing the performed results, but without statistical difference between them when averaging across the subject set. Furthermore, the bi-class accuracy of CWT-based vectors is comparable to that obtained by the best case of PSD-based extraction vectors, having a very similar ranking of individual performance.

**Table 1:** Performed bi-class accuracy within the MI segment using the whole electrode montage ($C{=}22$)

Subject	$\mu$	$\beta$	$\mu \cup \beta$	$\mu \cup 3\beta$
*PSD*				
A08T	*97.1* ± *3.5*	84.9 ± 6.9	93.3 ± 3.9	96.8 ± 3.9
A09T	92.1 ± 4.8	89.0 ± 6.1	96.5 ± 4.3	*96.6* ± *4.1*
A03T	91.1 ± 5.6	77.5 ± 6.4	91.2 ± 6.5	*91.3* ± *4.2*
A06T	80.0 ± 6.9	81.6 ± 5.5	82.5 ± 6.1	*86.7* ± *5.8*
A07T	83.8 ± 8.7	78.9 ± 4.3	82.0 ± 4.9	*85.1* ± *8.6*
A05T	76.2 ± 5.2	79.0 ± 6.9	80.8 ± 6.6	*84.0* ± *4.5*
A04T	*84.7* ± * 6.2*	79.3 ± 7.6	80.7 ± 7.5	83.7 ± 5.4
A01T	82.6 ± 3.4	80.5 ± 5.4	82.6 ± 5.6	*83.5* ± *7.0*
A02T	80.4 ± 6.8	77.8 ± 6.2	81.1 ± 5.3	*82.5* ± *6.4*
Average $h_u$	200	350	350	300
Average $a_c$	85.3 ± 5.7	80.9 ± 6.1	85.6 ± 5.6	*87.8* ± *5.5*
*CWT*				
A03T	*96.4* ± *3.6*	95.0 ± 4.6	95.0 ± 4.6	94.2 ± 2.9
A08T	*96.3* ± *4.9*	95.4 ± 5.1	94.0 ± 4.6	94.0 ± 5.6
A09T	94.0 ± 5.4	94.8 ± 5.6	*94.8* ± *4.2*	92.3 ± 5.9
A07T	85.7 ± 7.9	83.4 ± 6.8	86.4 ± 6.7	*87.4* ±*5.4*
A06T	83.9 ± 5.6	84.1 ± 5.1	86.7 ± 7.2	*86.7* ± *7.2*
A04T	*86.9* ± *7.7*	84.6 ± 8.4	85.4 ± 7.3	85.3 ± 7.2
A01T	83.2 ± 6.7	82.5 ± 5.1	*83.4* ± *5.5*	82.5 ± 3.9
A05T	79.8 ± 6.3	76.8 ± 9.0	79.1 ± 4.8	*82.2* ± *4.9*
A02T	81.8 ± 7.2	*84.0* ± *8.2*	83.8 ± 6.5	81.7 ± 7.2
Average $h_u$	350	400	250	250
Average $a_c$	87.5 ± 6.1	86.7 ± 6.4	*87.6* ± *5.7*	87.4 ± 5.6

The best figure achieved by each individual is marked in italics

**Fig. 2:**
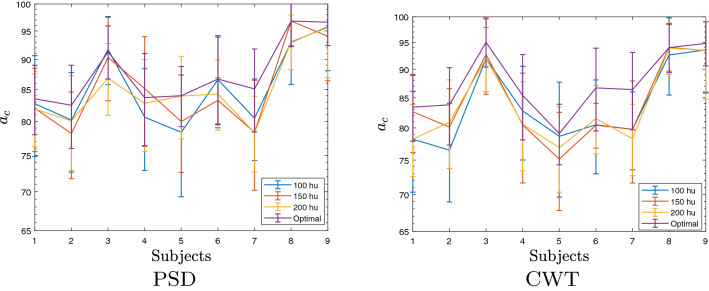
Dependence of CNN hidden units and the individual bi-class accuracy. Label “optimal” is the individually tuned CNN model

In terms of the tuned CNN parameters, their values averaged across the subject set show that the training scenario, achieving the best accuracy ($\mu {\cup } \beta _{{\text {low}}} {\cup } \beta _{{\text {med}}} {\cup } \beta _{{\text {high}}}$), demands from the PSD-based vectors more hidden units $h_u$ than in the case of CWT planes. A similar situation holds in the scenario $\mu {\cup }\beta$ that also performs high accuracy. When extracting the *t-f* vectors from a single rhythm ($\mu$ or $\beta$), the PDS-based representation demands less hidden units but achieves lower accuracy.

Figure 2 displays the dependency of CNN hidden units on the obtained bi-class accuracy. Compared to the best score achieved by the individually tuned value of $h_u$, the deterioration in performance is noticeable (nearly $5\%$) when decreasing the number of units in every trained CNN model. At the same time, the computational burden can reduce, on average, about a quarter time. Moreover, the variations in accuracy by changing the amount of $h_u$ indicate a similar complexity for both measured extraction approaches.

**Fig. 3:**
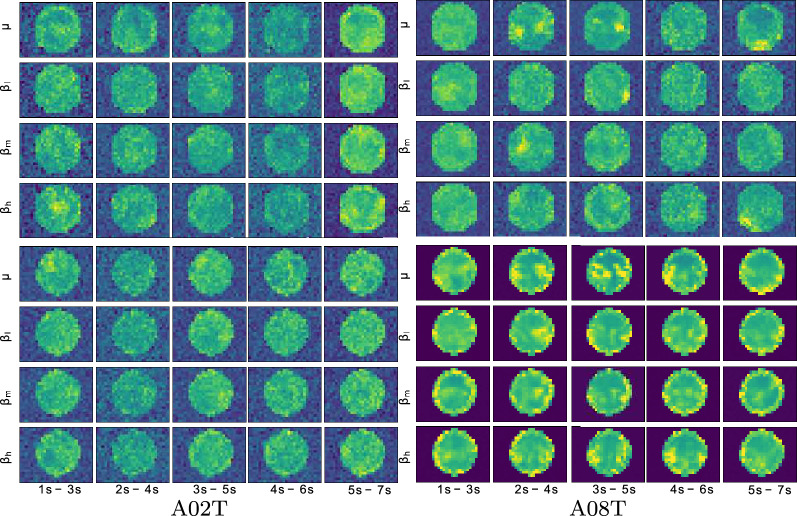
Reconstructed topographic maps for the bi-class experiment *D* including all bandwidths across the time domain in the best (A08T) and the worst (A02T) subjects, left and right column, respectively

Interpretability of brain areas activated by MI tasks. Intending to give the interpretability of the extracted input *t-f* vectors, we represent the feature mapping graphically (topoplot) to highlight the spatial distribution of the assessed discriminative ability. Each topoplot depicts the proposed assessment ${\varvec{\tilde{\varTheta }}} ({\varvec{\rho }}^{r,\varDelta f})$ computed in Eq. (6) in which we reconstruct the input feature image from the trained CNN weights to estimate the contribution of the electrodes, under the assumption that the higher the reconstructed weight, the more critical the discriminating strength between the electrodes. Of note, the interpolated values falling out of the electrode space are assumed as meaningless. This situation may arise because the network initializes the weight set with random values, including the background pixels. Therefore, the variability and reduced signal-to-noise rate result in false augmentation of background localities, as subjects reach low discrimination ability.

The top row of Fig. 3 displays the PSD-based spatial distribution reconstructed for the best training scenario (*D*) within each time segment. As seen, the topoplots of A02T (the worst individual) present the spectral bandwidths contributing much alike with values mainly spread all over space, including places outside of the electrode space. In addition, the contribution estimates are low and tend to be noisy. Another fact to mention is that brain activity notably increases within the last time segment, for which the MI activity is thought to have already vanished. By contrast, the best-achieving subject A08T has some relevant localities, which gather in places of either brain hemisphere and within the MI interval, fading at the time window $$ [4{-}6]$$ s.

In turn, the bottom row depicts the CWT-based topoplots assessed by the same training scenario (i.e., *D*), showing that the obtained spatial distribution of A02T still presents the spectral bandwidths that contribute similarly. However, several spatial clusters appear, and the amount of meaningless estimates decreases. Nevertheless, a notably enhanced topographic representation is performed by A08T, for which the CWT-based vectors result in values adequately accommodated within the electrode space, regardless of the window time. Furthermore, the contribution concentrates on the electrode neighborhoods clearly defined, changing over time. Thus, the $\mu$ rhythm shows that the sensorimotor electrodes contribute the most, being more evident their importance at the window $[3{-}5]$ s, right at the MI period.

**Fig. 4:**
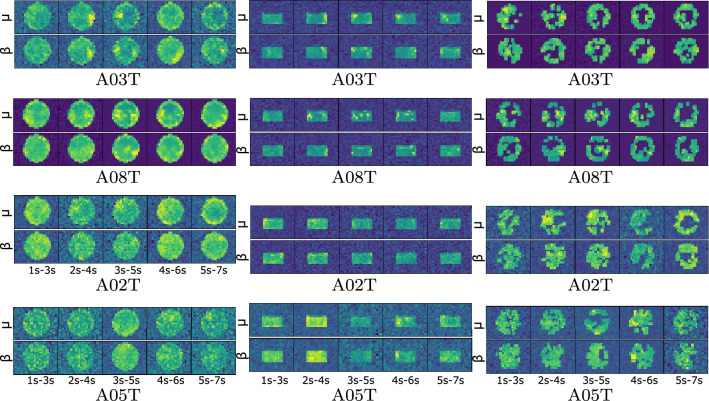
Individual relevance weights performed by the tuned CWT feature extraction for the bi-class scenario *C*: Without spatial dropping (left column), spatial dropping by removing all sensorimotor electrodes out (middle column), spatial dropping by excluding nonrelevant electrodes (right column)

Figure 4 (left column) displays the topoplots individually computed for the CWT-based feature extraction under the scenario C (i.e., $\mu {\cup }\beta$), showing that the brain activity tends to gather over some electrodes in most of the subjects. Also, the brain activity between neighboring time windows changes smoothly, at least in subjects performing high accuracy (i.e., A03T and A08T). As the discrimination ability of individuals decreases, the topographic representations become more blurred, meaning that the learned weights are still severely affected by the variability captured by EEG data. This situation is more visible in A05T (performing the worst) with much learning weights out of the scalp area, evidencing that the CNN model is likely to be overtrained.

Performance of spatial dropping strategies. Two approaches are evaluated—(i) removing all electrodes out of the sensorimotor area before training and validation, and (ii) thresholding the electrode contribution after training and validation.

The first spatial dropping strategy is implemented by simply including all electrodes belonging to the motor cortex region (that is, C3,9,10,11,C4,14,15,16,17,18), following the spatial electrode distribution reported by [40]. Figure 4 (middle column) depicts the estimated topoplots of the two best and worst-performing subjects, showing that the brain activity gathers more prominently over some lateral sensorimotor electrodes in most of the subjects. Moreover, the brain activity between neighboring time-windows changes smoothly with the highest contribution within the segments of MI ([$2{-}4$] and $[3{-}5]$ s). In the first couple of subjects (A08T and A03T), the contribution of either rhythm ($\mu$ or $\beta$) differs. Besides, the number of learning values out of the scalp is considerably smaller than in the previous case. Still, the topographic representations of the subjects with the worst accuracy (A02T and A05T) remain blurred.

**Table 2:** Bi-class accuracy of evaluated CNN training strategies, using the CWT-extracted vectors and either dropping strategy: CWT* with sensorimotor electrodes and CWT** with thresholding. In all compared cases, both sub-bands ($\mu$ and $\beta$) are included and the CNN parameters are tuned individually

Subjects	[41]	[42]	CWT	$\kappa$	CWT*	$\kappa$	CWT**	$\kappa$
A03T	88.2	91.7	95.0 ± 4.6	0.67	*96.4* ± *4.8*	0.92	95.0 ± 4.6	0.90
A09T	82.7	90.9	*94.8* ± *4.2*	0.68	93.1 ± 6.5	0.86	94.0 ± 6.3	0.88
A08T	91.8	92.3	$\underline{94.0\pm 4.6}$	0.90	*97.0*±*3.6*	0.94	94.7 ± 4.9	0.89
A06T	65.7	78.5	86.7 ± 7.2	0.71	84.9 ± 9.0	0.69	*86.7* ±* 7.2*	0.73
A07T	51.7	86.5	*86.4* ±* 6.7*	0.58	$\underline{81.9\pm 6.2}$	0.64	85.6 ± 9.3	0.71
A04T	53.9	80.4	85.4 ± 7.3	0.73	86.1 ± 7.5	0.72	*87.6* ± *5.0*	0.75
A02T	63.9	68.4	*83.8* ±* 6.5*	0.73	80.3 ± 6.2	0.61	81.7 ± 4.8	0.63
A01T	79.4	87.8	*83.4* ± *5.5*	0.88	81.1 ± 5.0	0.62	83.2 ± 3.9	0.66
A05T	54.9	88.9	*79.1* ±* 4.8*	0.90	78.3 ± 7.4	0.57	76.7 ± 6.9	0.53
Average	70.2	85.0 ± 7.4	*87.6* ± *5.7*	0.75	86.6 ± 6.2	0.73	87.4 ± 5.7	0.74

Concerning the second dropping strategy, Fig. 4 (right column) represents the thresholded values, showing the presence of several electrodes with a relevant contribution. Thus, the top pair of subjects holds the learned weights located on the lateral zones, having the highest contribution near the sensorimotor area with differentiated behavior between $\mu$ and $\beta$ rhythms. As expected, the central localities near the longitudinal fissure have zero-valued weights. However, as the individual performance decreases, the number of relevant electrodes increases due to the increased variability. Moreover, the variance of the captured EEG data for the worst-performing subjects is so strong that they have a distorted topoplot with values out of the scalp. Still, these subjects present relevant electrodes, unlike the previous approaches achieved.

Table 2 summarizes the bi-class performance achieved by each evaluated CNN-based framework, showing that every subject reaches a performance above $\sim 75\%$. All achieved accuracy scores are competitive with other values performed by CNN-based approaches recently presented for motor imagery classification (left and right hand). It is worth noting that the use of either spatial dropping strategy results in small degradation of classifier accuracy or $\kappa$ value.

Performance of three-class MI tasks. Further, we evaluate the proposal in a more complicated classification scenario, conducting testing for the following three-class discrimination framework of motor imagery tasks: left hand, right hand, and foot. Table 3 summarizes the classifier performance reported by two state-of-the-art approaches to the three-class discrimination, showing that the proposed approach provides very competitive outcomes (above 71%) and enhancing the accuracy of the low-performing subjects. One aspect to remark is that the values of multi-class accuracy and $\kappa$ tend to fall, compared to the bi-class scenario, partially because of the small database evaluated.

**Table 3:** Three-class accuracy of evaluated CNN training strategies using the CWT-extracted vectors for the considered strategies: without spatial dropping (CWT), spatial dropping by removing all sensorimotor electrodes out (CWT*), spatial dropping by excluding nonrelevant electrodes (CWT**)

Subjects	[43]	[44]	CWT	$\kappa$	CWT*	$\kappa$	CWT**	$\kappa$
A03T	*86.1*	76.7	83.6 ± 6.6	0.75	84.6 ± 8.3	0.76	82.0 ± 5.6	0.73
A07T	*83.6*	82.8	73.5 ± 9.5	0.59	73.9 ± 7.5	0.60	75.0 ± 7.4	0.63
A08T	63.1	*78.2*	73.0 ± 9.0	0.59	76.0 ± 9.5	0.64	75.1 ± 10.4	0.63
A01T	80.8	*84.1*	73.0 ± 6.6	0.59	73.4 ± 6.1	0.60	75.7 ± 5.5	0.63
A09T	54.3	*75.2*	72.2 ± 5.6	0.58	69.4 ± 5.7	0.58	73.0 ± 7.6	0.60
A04T	54.0	62.4	69.7 ± 5.4	0.47	69.7 ± 6.9	0.47	*69.9 ± 5.6*	0.47
A06T	51.3	51.8	68.3 ± 6.7	0.49	*68.8 ± 8.5*	0.52	68.4 ± 6.8	0.52
A02T	*71.2*	57.7	65.0 ± 5.5	0.44	58.9 ± 6.5	0.46	62.4 ± 8.8	0.46
A05T	52.2	48.1	*59.1 ± 4.2*	0.37	58.5 ± 5.6	0.36	57.5 ± 6.0	0.34
Average	66.2	68.5	70.8 ± 6.5	0.54	70.3 ± 7.0	0.55	*71.2 ± 7.0*	0.56

**Fig. 5:**
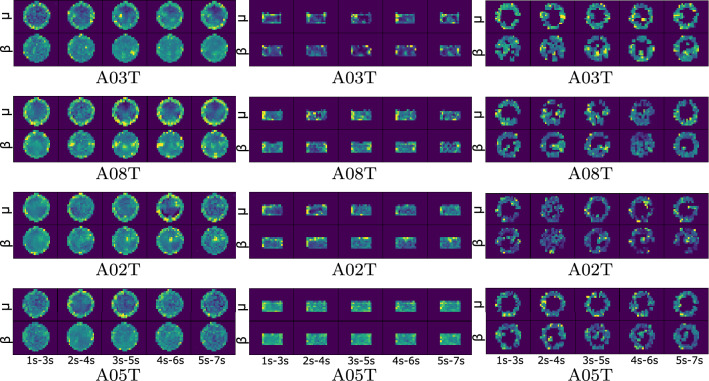
Relevance weights computed for representative individuals (best-performing A03T and A08T and worst-performing A02T and A05T) in terms of performed three-class accuracy for scenario *C*: without spatial dropping (left column), spatial dropping by weighing only all sensorimotor electrodes (middle column), spatial dropping by excluding nonrelevant electrodes (right column)

As in the binary classification task, we analyze the interpretability of brain areas activated by each MI task based on the reconstruction of the learned CNN weights. When assessed by the CWT-based feature extraction, Fig. 5 displays the reconstructed topoplots of scenario C, having a more distinct electrode contribution than in the bi-class case. If not using the spatial dropping, the dyad of the best-performing subjects shows an increase in neural activity within the motor imagery interval ($[3{-}5]$ s). However, this behavior is not evident in the worst-performing pair. Moreover, subject A02T has a response postponed to the segment ($[4{-}6]$ s).

In the next case of sensorimotor dropping, the middle column shows that the better the accuracy, the more compact the electrode contribution. Thus, the method assesses the motor cortex’s regular contribution through the whole record, regardless of the evoked activity. This kind of scattered representation implies high intrasubject variability. Then, the spatial dropping by excluding nonrelevant electrodes (right column) enhances the interpretation of the learned CNN weights, yielding a lower number of contributing electrodes, but more meaningful.

Lastly, we evaluate the significance of learning CNN weights in terms of the disagreement of performing the individual accuracy, using the considered dropping strategies: spatial dropping by weighing only all sensorimotor electrodes (CWT*) and spatial dropping by excluding nonrelevant electrodes (CWT**). To this, we conduct the paired Welch’s t-test, employing the scores achieved on the cross-validation folds and holding a significant level of a *p-value*$<0.05$. In this case, the non-rejection of the null hypothesis (identical average scores) is the desired behavior to prove that our relevance approaches (CWT* and CWT**) do not differ from CWT (without spatial dropping). Table 2 shows that only a couple of subjects (namely, A08T with $p = 0.159$ and A07T with $p = 0.133$) that are underlined are close to $p<0.15$. In turn, Table 3 presents a confident difference in performance for subject A02T (underlined subject) with a $p = 0.039$. This result may be explained since A02T reports a low-performance with high variability along the folds. Hence, the sensorimotor region is not sufficient to code discriminant information about this subject.

## Discussion and concluding remarks

We present an approach using CNN models to improve the interpretability of spatial contribution in discriminating between MI tasks, preserving an adequate classification accuracy. The results obtained for BCI Dataset 2a prove that the proposed deep learning framework allows improving accuracy along with revealing the electrodes with higher spatial relevance. Nevertheless, the following aspects are to be regarded in the framework implementation:

Feature extraction of t-f vectors. For each estimated source, the *t-f* sets are extracted within each time window, generating an image containing temporal, spectral, and spatial information. Intending to deal with the nonstationary EEG nature, we evaluate the extraction of *t-f* patterns from the FFT-based periodogram and continuous wavelet transform. Then, all extracted *t-f* feature patterns are further interpolated to obtain the spatial distribution of activated brain areas through topographic maps. We obtain that both approaches are similar in terms of providing classifier performance and the complexity of implementing CNN models. Besides, we evaluate four combining scenarios of $\mu$ and $\beta$ rhythms, which differently influence the achieved accuracy. In the case of PSD estimates, only the inclusion of detailed information from three $\beta$ sub-bands together with $\mu$ waveform provides the best system accuracy. By contrast, the CWT-based feature set gives high accuracy scores regardless of the evaluated sub-band combination. This result may be explained by the fact that CWT is more suitable for the decomposition of nonstationary data.

Nonetheless, the CWT-based vectors are preferable for interpretation purposes because the learned weights gather around electrode neighborhoods, forming more clearly defined spatialities with relevant neural activity. Moreover, the CWT-based weights smoothly change over time following the implemented MI paradigm timing. One more aspect of highlighting is that the learned weights are less sensitive to the overtraining effect.

Spatial interpretability of activated MI responses. Another aspect to remark is the dropout algorithm that CNN models include. Their high number of parameters makes them particularly prone to over-fitting, demanding the use of regularization methods. In addition, neural network training or inference can involve randomly modifying parameters [45]. To cope with this issue, the spatial dropout algorithm can withdraw an entire feature map across a channel since adjacent pixels are highly correlated to the dropped pixels [46, 47].

Relying on the fact that the interpretation of evoked brain zones can be performed by preserving spatial information in input multi-spectral images, we evaluate two spatial dropping strategies to promote discarding of irrelevant image details: including just the sensorimotor electrodes, and thresholding of the electrode contribution. Although the number learned values out the scalp decreases considerably in the former strategy, the topographic representations of subjects having low accuracy are still blurred, hindering the interpretation of analyzed brain activity. The use of full-set EEG electrodes has been already reported as difficult to achieve in practical MI applications, suggesting that the performance of CNN models can improve with fewer electrodes, which cover the motor cortex and sensorimotor cortex [48]. The obtained results show that the thresholding strategy is desirable since the highest contribution clusters over the sensorimotor area with differentiated behavior between $\mu$ and $\beta$ bands. However, the high EEG data variability captured by the worst-performing subjects may still produce distorted topoplots with values out of the scalp, making difficult their understanding.

Evaluated CNN architecture for MI discrimination. The first design consideration is the number of convolutional layers, together with the type of end classifier. In MI tasks, 70% of CNN models use a rectified linear unit (ReLU) as the layer’s activation function, while the vast majority of classifier fully connected layers employ a softmax activation function [49]. The proposed network relies on the *Wide&Deep* architecture for handling multiple inputs to learn deep patterns under simple rules. With the purpose of increasing the neurophysiological reliability of feature interpretation, the *Classifier Block* includes batch normalization applied to the convolutional outputs before and after the fully connected layer FC7, improving the performance on unseen examples [50]. We also use the Elastic Net regularization technique through the parameters ($l_1$, $l_2$) for preventing over-fitting by penalizing a model with large weights.

Spatial dropping of multi-class settings. The spatial dropping algorithm is evaluated in two labeled scenarios of MI tasks: bi-class and three-class, resulting in meaningful topographic representations and performing values of accuracy very competitive with the results reported by similar CNN-based architectures. However, the achieved values of multi-class accuracy and $\kappa$ tend to fall, compared to the bi-class scenario. This behavior can be partially explained by the small database evaluated and the reduced set of scalp electrodes.

However, some restrictions are to be mentioned: the first limitation to enhance the performance of the evaluated CNN architecture is the small size of the examined dataset that holds just nine subjects with very different variability [51]. As a result, the deterioration in performance is noticeable (nearly $5\%$) when decreasing the number of units in each individual trained CNN model. Moreover, the small data issue restricts the application of robust approaches in deep learning like augmentation or transfer learning, causing over-fitting. Another concern is the adequate sampling of the potential scalp field for the topographic analysis that requires a large number of electrodes [52].

As future work, to enhance the impact of tested Deep Learning models, we plan to employ datasets that hold more labeled MI tasks, fusing CNNs with different characteristics and architectures is also to be considered to learn more complex relationships between spatial patterns and extracted *t-f* representations, making the learned CNN weights more accessible to interpret [53, 54].

## Data Availability

Publicly available datasets were analyzed in this study. This data can be found here:http://www.bbci.de/competition/iv/#download.

## Footnotes

1 *BCI Competition IV*, publicly available at www.bbci.de/competition/iv/.

2 function topoplot() in *EEGLAB* toolbox.

## References

[1] Cannard C, Brandmeyer T, Wahbeh H, Delorme A (2020) Chapter 16-Self-health monitoring and wearable neurotechnologies. In: Ramsey NF, Millán JDR (eds) Brain-Computer Interfaces. Handbook of Clinical Neurology, vol.168. Elsevier, pp 207–232 10.1016/B978-0-444-63934-9.00016-0 32164854

[2] Xu M, Wei Z, Ming D (2020). Research advancements of motor imagery for motor function recovery after stroke. Sheng wu yi xue Gong Cheng xue za zhi= Journal of Biomedical Engineering= Shengwu Yixue Gongchengxue Zazhi.

[3] Guillot A, Debarnot U (2019). Benefits of motor imagery for human space flight: a brief review of current knowledge and future applications. Front Physiol.

[4] Pillette L, Jeunet C, Nkambou R, N’Kaoua B, Lotte F (2019) Towards artificial learning companions for mental imagery-based brain-computer interfaces. CoRR, arXiv:abs/1905.09658

[5] Frau-Meigs D (2007) Media Education. A Kit for Teachers, Students, Parents and Professionals. UNESCO

[6] Marchesotti S, Bassolino M, Serino A, Bleuler H, Blanke O (2016). Quantifying the role of motor imagery in brain-machine interfaces. Sci Rep.

[7] Rim B, Sung N, Min S, Hong M (2020). Deep learning in physiological signal data: a survey. Sensors.

[8] Sakhavi S, Guan C, Yan S (2018). Learning temporal information for brain-computer interface using convolutional neural networks. IEEE Transactions on Neural Networks and Learning Systems.

[9] Zemouri R, Zerhouni N, Racoceanu D (2019). Deep learning in the biomedical applications: recent and future status. Appl Sci.

[10] Plis S, Hjelm D, Salakhutdinov R, Allen E, Bockholt H, Long J, Johnson H, Paulsen J, Turner J, Calhoun V (2014). Deep learning for neuroimaging: a validation study. Front Neurosci.

[11] Wu H, Niu Y, Li F, Li Y, Fu B, Shi G, Dong M (2019). A parallel multiscale filter bank convolutional neural networks for motor imagery EEG classification. Front Neurosci.

[12] Amin S, Alsulaiman M, Muhammad G, Bencherif M, Hossain M (2019). Multilevel weighted feature fusion using convolutional neural networks for EEG motor imagery classification. IEEE Access.

[13] Ortiz-Echeverri CJ, Salazar-Colores S, Rodrí-guez-Reséndiz J, Gómez-Loenzo R (2019). A new approach for motor imagery classification based on sorted blind source separation, continuous wavelet transform, and convolutional neural network. Sensors.

[14] Guan C, Tih-Shih L, Cuntai G, Fung S, Shuen D, Cheung Y, Teng S, Zhang H, Krishnan K (2010). Effectiveness of a brain-computer interface based programme for the treatment of adhd: a pilot study. Psychopharmacol Bull.

[15] Doborjeh M, Kasabov N, Doborjeh Z (2017). Evolving, dynamic clustering of spatio/spectro-temporal data in 3d spiking neural network models and a case study on EEG data. Evolv Syst.

[16] Yang H, Sakhavi S, Ang KK, Guan C (2015) On the use of convolutional neural networks and augmented csp features for multi-class motor imagery of EEG signals classification. In: 2015 37th Annual International Conference of the IEEE Engineering in Medicine and Biology Society (EMBC), pp 2620–2623 10.1109/EMBC.2015.7318929 26736829

[17] Sakhavi S, Guan C, Yan S (2015) Parallel convolutional-linear neural network for motor imagery classification. In 2015 23rd European Signal Processing Conference (EUSIPCO), pp 2736–2740

[18] Taheri M, Ezoji 
S, Sakhaei SM (2020). Convolutional neural network based features for motor imagery EEG signals classification in brain-computer interface system. SN Appl Sci.

[19] Tang X, Li W, Li X, Ma W, Dang X (2020). Motor imagery EEG recognition based on conditional optimization empirical mode decomposition and multi-scale convolutional neural network. Expert Syst Appl.

[20] Zhang J, Yan C, Gong X (2017) Deep convolutional neural network for decoding motor imagery based brain computer interface. In 2017 IEEE International Conference on Signal Processing, Communications and Computing (ICSPCC), pp 1–5

[21] Uktveris T, Jusas V (2017). Application of convolutional neural networks to four-class motor imagery classification problem. ITC.

[22] Lee HK, Choi Y (2018) A convolution neural networks scheme for classification of motor imagery EEG based on wavelet time-frequecy image. In: 2018 International Conference on Information Networking (ICOIN), pp 906–909

[23] Yang J, Yao S, Wang J (2018). Deep fusion feature learning network for MI-EEG classification. IEEE Access.

[24] Petrichella S, Vollere L, Ferreri F, Guerra A, Maatta S, Kononen M, Di Lazzaro V, Iannello G (2016) Channel interpolation in tms-EEG: a quantitative study towards an accurate topographical representation. Conference proceedings: Annual International Conference of the IEEE Engineering in Medicine and Biology Society. IEEE Engineering in Medicine and Biology Society. Annual Conference, 2016:989–992 10.1109/EMBC.2016.7590868 28268490

[25] Tabar Y, Halici U (2017). A novel deep learning approach for classification of EEG motor imagery signals. J Neural Eng.

[26] Thodoroff P, Pineau J, Lim A (2016) Learning robust features using deep learning for automatic seizure detection. CoRR. arXiv:1608.00220

[27] Xu G, Shen X, Chen S, Zong Y, Zhang C, Yue H, Liu M, Chen F, Che W (2019). A deep transfer convolutional neural network framework for EEG signal classification. IEEE Access.

[28] D’Souza RN, Huang P-Y, Yeh F-C (2020) Structural analysis and optimization of convolutional neural networks with a small sample size. Sci Rep 10(1):1–13 10.1038/s41598-020-57866-2 PMC6972775 31965034

[29] Dai M, Zheng D, Na R, Wang S, Zhang S (2019) EEG classification of motor imagery using a novel deep learning framework. Sensors 19(3):551 10.3390/s19030551 PMC6387242 30699946

[30] Rong Y, Wu X, Zhang Y (2020) Classification of motor imagery electroencephalography signals using continuous small convolutional neural network. Int J Imaging Syst Technol 30(3):653–659

[31] Zhao X, Zhang H, Zhu G, You F, Kuang S, Sun L (2019). A multi-branch 3d convolutional neural network for EEG-based motor imagery classification. IEEE Trans Neural Syst Rehab Eng.

[32] Bashivan P, Rish I, Yeasin M, Codella N (2016) Learning representations from EEG with deep recurrent-convolutional neural networks. CoRR, arXiv:abs/1511.06448

[33] McFarland D, Miner L, Vaughan T, Wolpaw J (2004). Mu and beta rhythm topographies during motor imagery and actual movements. Brain Topogr.

[34] Delorme A, Makeig S (2004). EEGLAB: an open source toolbox for analysis of single-trial EEG dynamics including independent component analysis. J Neurosci Methods.

[35] Alvarez-Meza AM, Velasquez-Martinez LF, Castellanos-Dominguez G (2015). Time-series discrimination using feature relevance analysis in motor imagery classification. Neurocomputing.

[36] Cheng H, Koc L, Harmsen J, Shaked T, Chandra T, Aradhye H, Anderson G, Corrado G, Chai W, Ispir M, Anil R, Haque Z, Hong L, Jain V, Liu X, Shah H (2016) Wide & deep learning for recommender systems. Association for Computing Machinery, New York, p 7–10

[37] Ide H, Kurita T (2017) Improvement of learning for CNN with ReLU activation by sparse regularization. In: 2017 International Joint Conference on Neural Networks (IJCNN), pp 2684–2691

[38] Lawhern V, Solon A, Waytowich N, Gordon S, Hung C, Lance B (2018). EEGNet: a compact convolutional neural network for EEG-based brain–computer interfaces. J Neural Eng.

[39] Li F, He F, Wang F, Zhang D, Xia Y, Li X (2020). A novel simplified convolutional neural network classification algorithm of motor imagery EEG signals based on deep learning. Appl Sci.

[40] Brunner C, Leeb R, Müller-Putz G, Schlögl A, Pfurtscheller G (2008) BCI competition 2008–graz data set A. Institute for Knowledge Discovery (Laboratory of Brain-Computer Interfaces), Graz University of Technology, vol 16

[41] Shahtalebi S, Asif A, Mohammadi A (2020) Siamese neural networks for EEG-based brain-computer interfaces. ArXiv. arXiv:2002.00904 10.1109/EMBC44109.2020.9176001 33018023

[42] Olivas-Padilla B, Chacon-Murguia M (2019). Classification of multiple motor imagery using deep convolutional neural networks and spatial filters. Appl Soft Comput.

[43] Zhou B, Wu X, Zhang L, Lv Z (2014). Guo X (2014) Robust spatial filters on three-class motor imagery EEG data using independent component analysis. J Biosci Med.

[44] Li B, Yang B, Guan C, Hu C (2019) Three-class motor imagery classification based on fbcsp combined with voting mechanism. In: 2019 IEEE International Conference on Computational Intelligence and Virtual Environments for Measurement Systems and Applications (CIVEMSA), pp 1–4

[45] Labach A, Salehinejad H, Valaee S (2019) Survey of dropout methods for deep neural networks. CoRR. arXiv:1904.13310

[46] Thompson M (2019). Critiquing the concept of bci illiteracy. Sci Eng Ethics.

[47] Park S, Kwak N (2017) Analysis on the dropout effect in convolutional neural networks. In: Lai S-H, Lepetit V, Nishino K, Sato Y (eds) Computer Vision – ACCV 2016, Springer International Publishing, Cham, pp 189–204

[48] Zhao X, Zhang H, Zhu G, You F, Kuang S, Sun L (2019). A multi-branch 3d convolutional neural network for EEG-based motor imagery classification. IEEE Trans Neural Syst Rehab Eng.

[49] Craik A, Kilicarslan A, Contreras-Vidal JL (2019) Classification and transfer learning of EEG during a kinesthetic motor imagery task using deep convolutional neural networks. In: 2019 41st Annual International Conference of the IEEE Engineering in Medicine and Biology Society (EMBC), pp 3046–3049 10.1109/EMBC.2019.8857575 31946530

[50] Borra D, Fantozzi S, Magosso E (2020). Interpretable and lightweight convolutional neural network for EEG decoding: application to movement execution and imagination. Neural Netw.

[51] Collazos-Huertas D, Caicedo-Acosta J, Castaño-Duque G, Acosta-Medina C (2020). Enhanced multiple instance representation using time-frequency atoms in motor imagery classification. Front Neurosci.

[52] Michel C (2019) Chapter 12 - high-resolution EEG. In Kerry H. Levin and Patrick Chauvel, editors, Clinical Neurophysiology: Basis and Technical Aspects, volume 160 of Handbook of Clinical Neurology, pp 185 – 201. Elsevier 10.1016/B978-0-444-64032-1.00012-6 31277847

[53] Amin S, Alsulaiman M, Muhammad G, Mekhtiche M, Hossain M (2019). Deep learning for EEG motor imagery classification based on multi-layer CNNS feature fusion. Future Gener Comput Syst.

[54] Wang Z, Cao L, Zhang Z, Gong X, Sun Y, Wang H (2018). Short time Fourier transformation and deep neural networks for motor imagery brain computer interface recognition. Concurr Comput.

